# Efficient digest of high-throughput sequencing data in a reproducible report

**DOI:** 10.1186/1471-2105-14-S11-S3

**Published:** 2013-09-13

**Authors:** Zhe Zhang, Jeremy Leipzig, Ariella Sasson, Angela M Yu, Juan C Perin, Hongbo M Xie, Mahdi Sarmady, Patrick V Warren, Peter S White

**Affiliations:** 1 Center for Biomedical Informatics, The Children's Hospital of Philadelphia, PA, USA; 2 Division of Oncology, The Children's Hospital of Philadelphia, PA, USA; 3 Department of Pediatrics, Perelman School of Medicine, University of Pennsylvania, PA, USA

## Abstract

**Background:**

High-throughput sequencing (HTS) technologies are spearheading the accelerated development of biomedical research. Processing and summarizing the large amount of data generated by HTS presents a non-trivial challenge to bioinformatics. A commonly adopted standard is to store sequencing reads aligned to a reference genome in SAM (Sequence Alignment/Map) or BAM (Binary Alignment/Map) files. Quality control of SAM/BAM files is a critical checkpoint before downstream analysis. The goal of the current project is to facilitate and standardize this process.

**Results:**

We developed bamchop, a robust program to efficiently summarize key statistical metrics of HTS data stored in BAM files, and to visually present the results in a formatted report. The report documents information about various aspects of HTS data, such as sequencing quality, mapping to a reference genome, sequencing coverage, and base frequency. Bamchop uses the R language and Bioconductor packages to calculate statistical matrices and the Sweave utility and associated LaTeX markup for documentation. Bamchop's efficiency and robustness were tested on BAM files generated by local sequencing facilities and the 1000 Genomes Project. Source code, instruction and example reports of bamchop are freely available from https://github.com/CBMi-BiG/bamchop.

**Conclusions:**

Bamchop enables biomedical researchers to quickly and rigorously evaluate HTS data by providing a convenient synopsis and user-friendly reports.

## Background

The development of high-throughput sequencing (HTS) technologies has lead to major biomedical discoveries in recent years [1-3]. The power of these technologies comes from the repeated sequencing of genomic regions of interest, such as exons [4] and protein binding sites [5], and requires processing millions of sequencing reads contained within raw data files sized between several hundred megabytes to over twenty-five gigabytes [6]. Reads are typically mapped to a reference genome via specifically designed alignment programs [7]. Mapped read counts are subsequently used for quantitative analysis, such as allele frequency of DNA mutations [8], abundance of mRNA in a tissue of interest [9], and frequency of protein-DNA binding [10].

The large amount of HTS data challenges the development of more efficient, robust, and reproducible data analysis workflows. One of the most successful efforts to standardize HTS workflow was the development of the Sequence Alignment/Map (SAM) format for the storage of aligned sequencing reads, along with a corresponding set of utility programs operating on SAM files [11]. SAM and its more practically utilized binary companion, BAM (Binary Alignment/Map), have been generally accepted as a standard to store and exchange aligned reads by the genomics community, including sequencing facilities and large-scale HTS projects such as the 1000 Genomes [12] and ENCODE [13] projects. BAM files can be further sorted and indexed to support random access to reads mapped to any genomic location.

Version 1.4 of the SAM format has eleven mandatory fields that can be classified into two categories. Each of the per read fields represents one aspect of each aligned read with a single value. For example, the "POS" field stores the mapped location of a read within a reference sequence, and the "MAPQ" field corresponds to a score assigned by the alignment program to indicate the confidence of the mapping. Per base fields "SEQ" and "QUAL" respectively record the base calls and sequencing quality scores of all bases in each read. These two fields account for the majority of the size of SAM/BAM files. The "CIGAR" field is a special case. It uses a compact character string to depict the actual base pair alignment. For example, "75M" means there are 75 bases of the read aligned to the reference sequence without gap, whereas "20M1D55M" means there is a single base deletion between the twentieth and twenty-first bases of all 75 bases.

The creation of BAM files is a milestone that typically marks the transition from raw data generation/processing to specific downstream analysis of HTS data. Once provided with the BAM files, researchers often need to evaluate data quality and identify potential issues that might affect downstream analysis. Examples of common questions are whether the sequencing quality and depth are sufficient to support robust quantitative analysis, and which lessons are learned from the current data to optimize future experiments. Close inspection of the BAM files is necessary to address these needs.

Programs systematically evaluating HTS data are available but scarce. FastQC summarizes sequencing quality, nucleic acid bias, and other information about the sequencing reads themselves, but does not provide information related to read alignment and sequencing coverage [14]. RNA-SeQC is used to specifically summarize read count, coverage, and expression correlation of RNA-seq data, but is not applicable to other types of HTS data [15].

The R programming language provides an ideal platform for summarizing HTS data due to its extensive functionality in scientific computation and data illustration [16], as well as its support of bioinformatics data analysis through the Bioconductor project [17]. Sweave is a framework that integrates R code within LaTeX documents [18]. Sweave enables the insertion of dynamic content, such as project-specific outputs, in a preformatted template, enabling highly efficient reporting of data analysis results.

To address the need for a more comprehensive evaluation of HTS data, we used the R framework to create an automated assessment method that was implemented in the bamchop program. Bamchop retrieves information from BAM files and reports a series of statistical matrices related to assorted aspect of HTS data.

## Implementation

### Hardware and software

Bamchop was developed and tested on a Unix server (Dell R610) with two 6-core Xeon CPUs and 192 GB of RAM using the R environment (version 2.15.0). The software requires several extension R packages (full list available via project website) that are mostly available through the Bioconductor project. For example, the Rsamtools package is an essential element of the program by providing the interface to access BAM files. Bamchop also depends on a TeX installation to generate PDF formatted documents from LaTeX intermediates.

### Inputs

Bamchop has a very simple command line user interface. The program requires only two inputs: a BAM file and an R data object containing information about the reference genome to which the sequencing reads were mapped. Examples of such information are base frequency of each chromosome and exon/intron locations. Bamchop does not require any information about how the BAM file was generated, which makes it applicable to any experimental protocol, sequencing platform, or alignment program. However, bamchop does require the BAM file to be sorted and indexed.

### System architecture

The major components of bamchop include a "main" program, a set of utility functions used to calculate statistics, a set of plotting functions used to generate illustrations, a database of genome-related information, and a Sweave reporting template.

**Figure 1:**
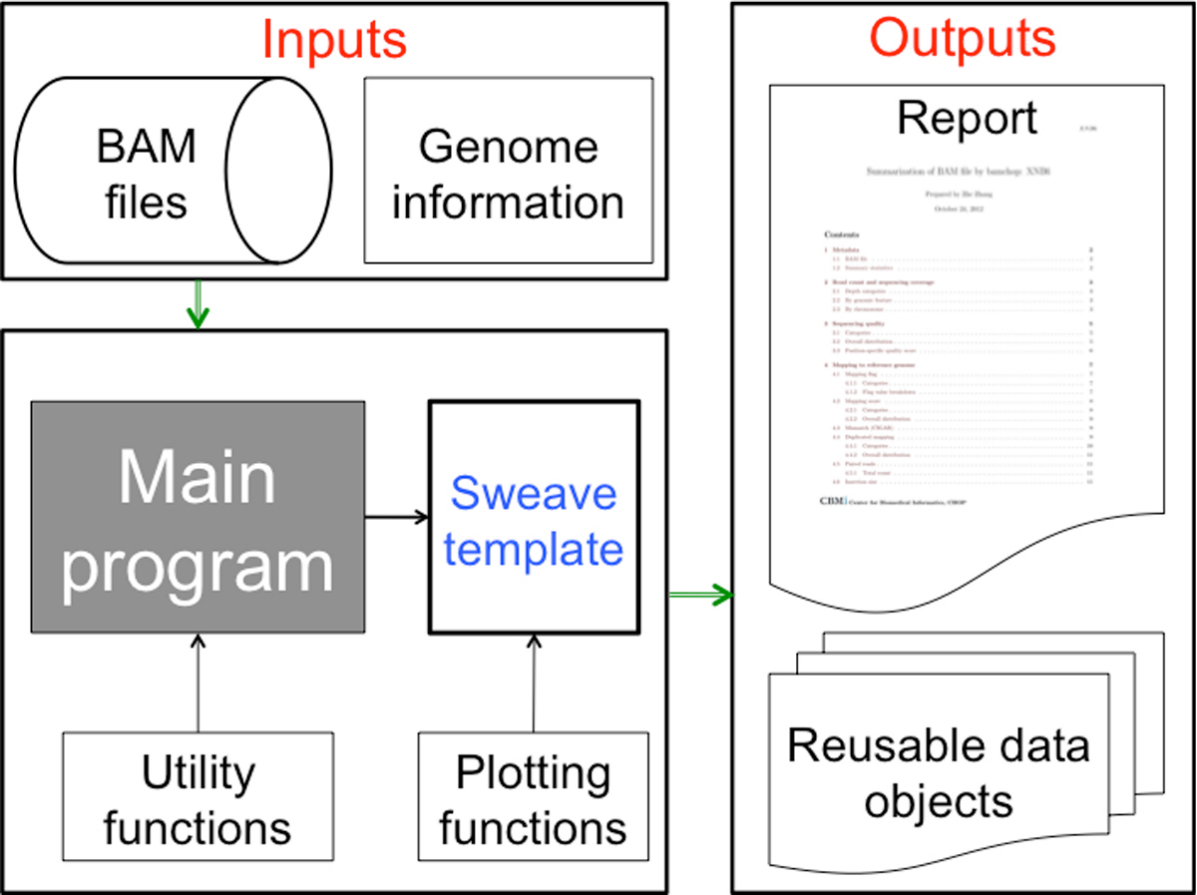
**System architecture of bamchop program**.

The workflow of a bamchop run is described in Figure 1. First, the scanBam() function implemented by the Rsamtools package loads mapping locations of all reads and all SAM fields of a randomly selected subset of reads from a BAM file. The latter is a necessary compromise to reduce system requirements and runtime (discussed below). An R data object storing the genome metadata will also be loaded. Bamchop includes pre-compiled information related to human and mouse genomes (hg19, hg18 and mm9) that are stored in an internal database, but users can also prepare their own genome/build metadata using a utility function. Once the main program loads the input data into the R environment, it calls a series of utility functions that statistically summarizes various aspects of the HTS data and saves the results in a structured "bamchop" data object. The object is then passed to a Sweave template to generate illustrations in a LaTeX document. This document is then converted to a PDF file as the final step.

The overall architecture of bamchop is straightforward and flexible. The main program and the utility functions are responsible for the generation of statistical matrices by performing most of the computational tasks, while the Sweave template transforms results into a report. These two layers communicate through a single "bamchop" object. This object can be saved and reused when the Sweave template is updated. Furthermore, its contents can be extended to include additional information, such as strand-specific sequencing depth, for downstream analysis without affecting the generation of the report.

### Outputs

The primary output of bamchop is an indexed PDF file with several sections corresponding to assorted aspects of the HTS data. The detailed contents of this report are described in the Results. Optionally, metadata and statistical results generated during the process can be written to the disk for re-use.

## Results and discussion

### Estimate statistics with random subset of sequencing reads

The large and ever-increasing size of HTS data will be a continuous challenge to any hardware and software. Loading multi-gigabyte BAM files into R environment is a time-consuming task even for powerful server systems. To explore methods of alleviating potential data load bottlenecks, we investigated whether a subset of randomly selected sequencing reads serves as a precise proxy for generating global statistics, especially those based on the per-base SAM fields.

**Figure 2:**
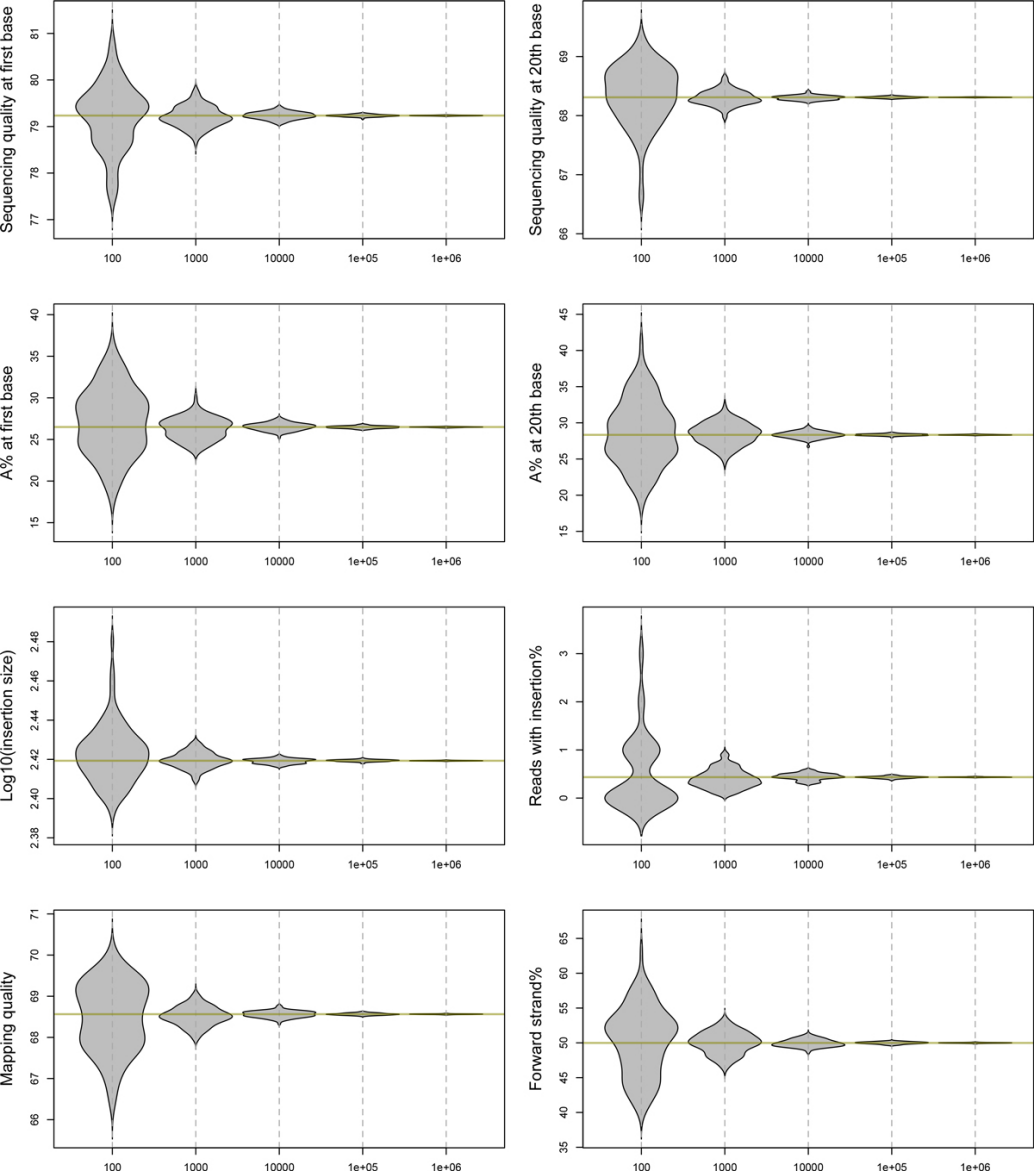
**Estimation of summary statistics by randomly selected sequencing reads**. The x-axis indicates the number of reads selected from a BAM file while the y-axis represents the values of eight summary statistics estimated using the selected reads. Each "violin" in the plots represents the distribution of estimated statistics based on 100 resamplings while the horizontal lines correspond to the global averages.

A resampling procedure was performed to randomly select 100 to 1 million aligned reads and import all SAM fields of these reads from a BAM file. A set of summary statistics were compiled from each of these random subsets, such as position-specific sequencing quality, base frequency, insertion size of paired reads, and mapping quality. This procedure was repeated 100 times. The distributions of summary statistics obtained from these repeats are displayed in Figure 2. The results indicate that for random subsets of 10^5 ^or more reads, the estimation of global statistics closely approached the true values. For example, the iterated estimates of the average insertion size of paired-end reads ranged between 261.8 and 263.4 bp when 10^5 ^random reads were used; whereas the global average of a total of over 300 million reads was 262.6 bp. We concluded that 10^5 ^reads are sufficient to precisely and consistently reproduce global statistics. Conversely, mapping locations of all reads are imported from BAM files into bamchop because they are relatively lightweight and required by most downstream analyses. The storage of minimal mapping information (chromosome, position, and strand) takes less than one gigabyte of memory for 100 million reads.

### Report content

**Figure 3:**
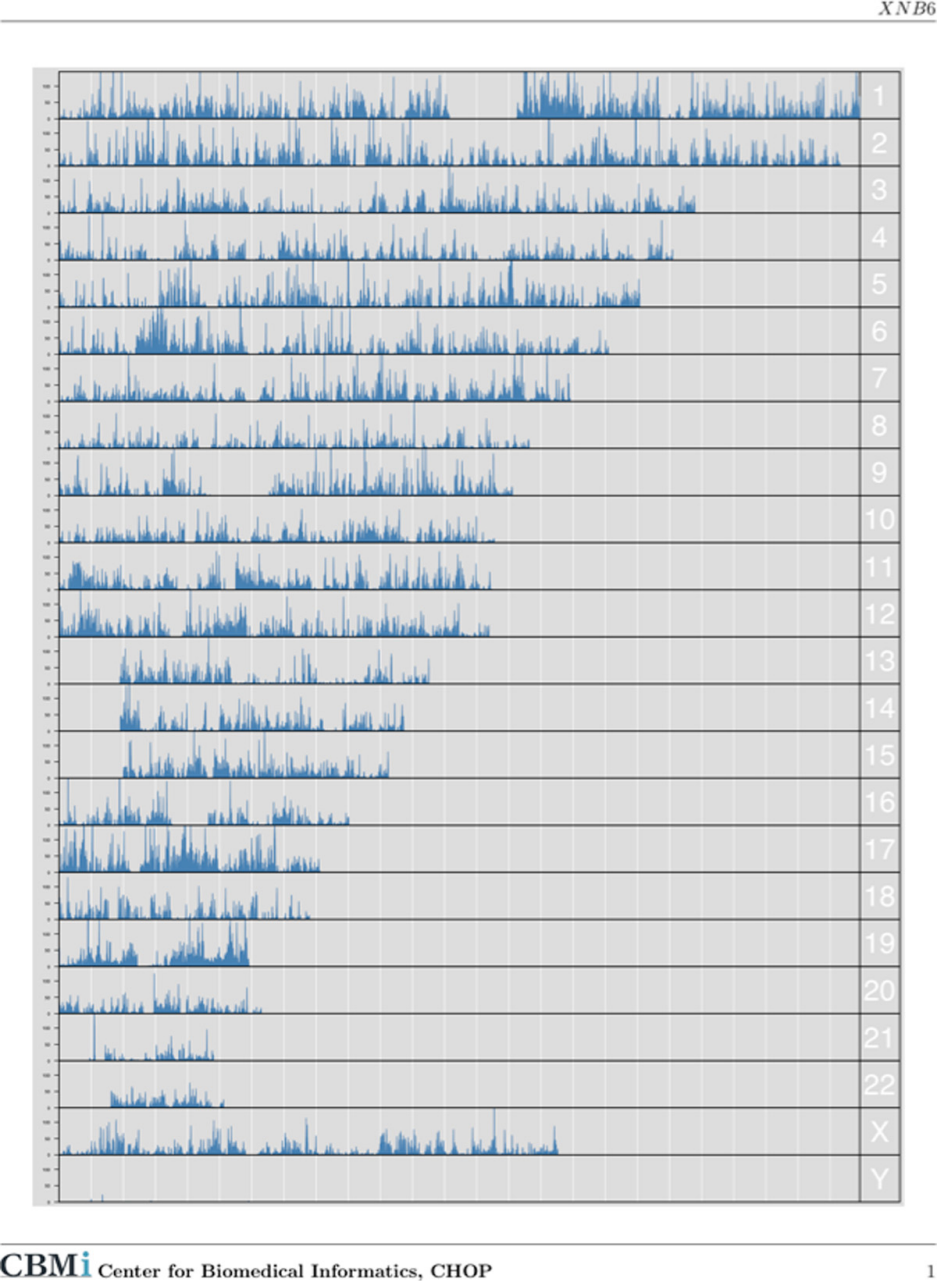
**This graphic index represents the sequencing depth along chromosomes**. It can be used to quickly identify large regions with extraordinarily high or low depth.

**Figure 4:**
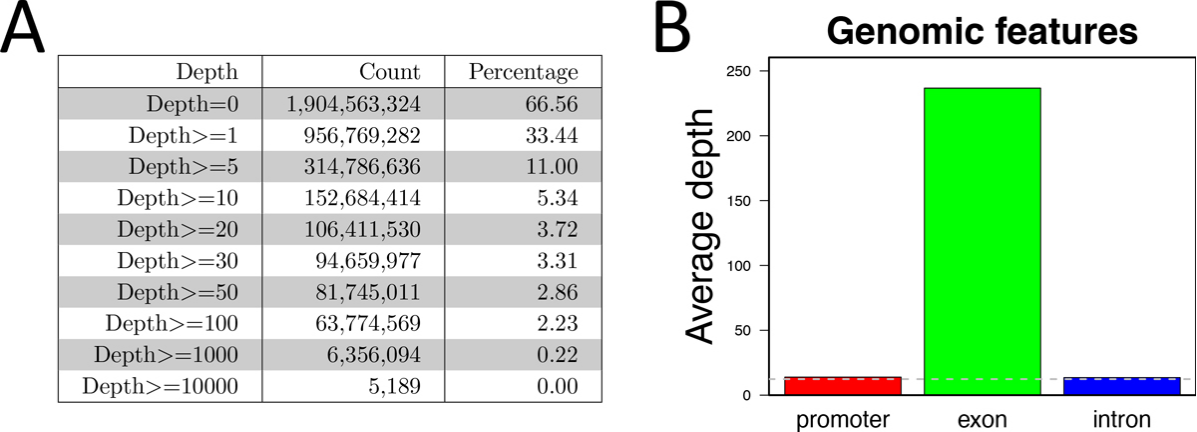
**Sequencing depth of an exome sequencing sample**. (A) The number and percentage of genomic locations with sequencing depth exceeding given values. (B) Mean sequencing depth of different genomic features. As expected for this exome sample, the exons have much higher mean depth than the other regions.

**Figure 5:**
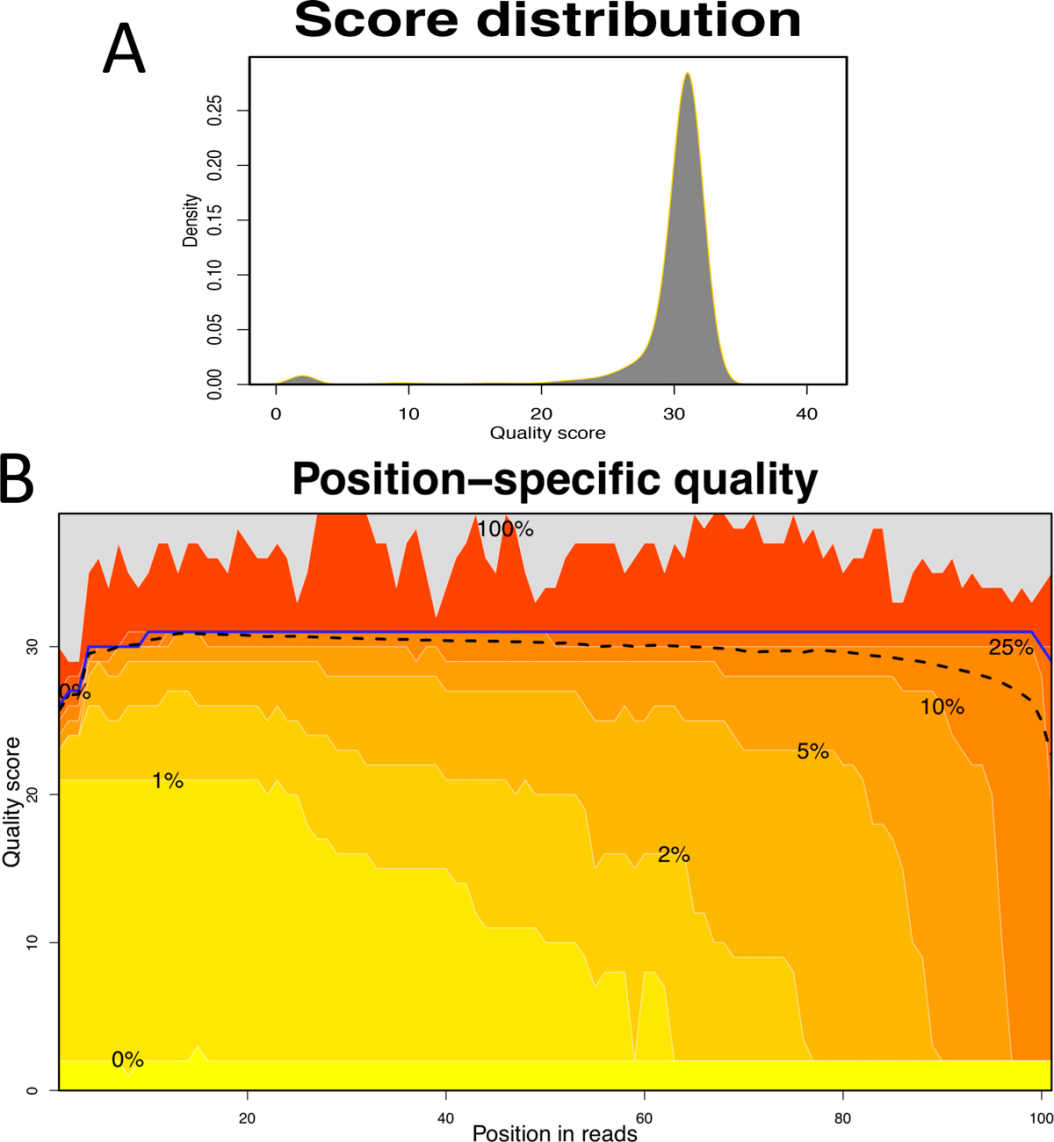
**Sequencing quality**. (A) Global distribution of single-base quality scores. An overall high quality of HTS data is suggested as most of the bases were scored around 30 (p = 0.001). (B) Heat map of sequencing quality as a function of read position. The blue solid line and black dashed line indicate the position-specific medians and means of scores, respectively. This figure suggests that the overall sequencing quality of this sample was substantially reduced after 80 bases.

**Figure 6:**
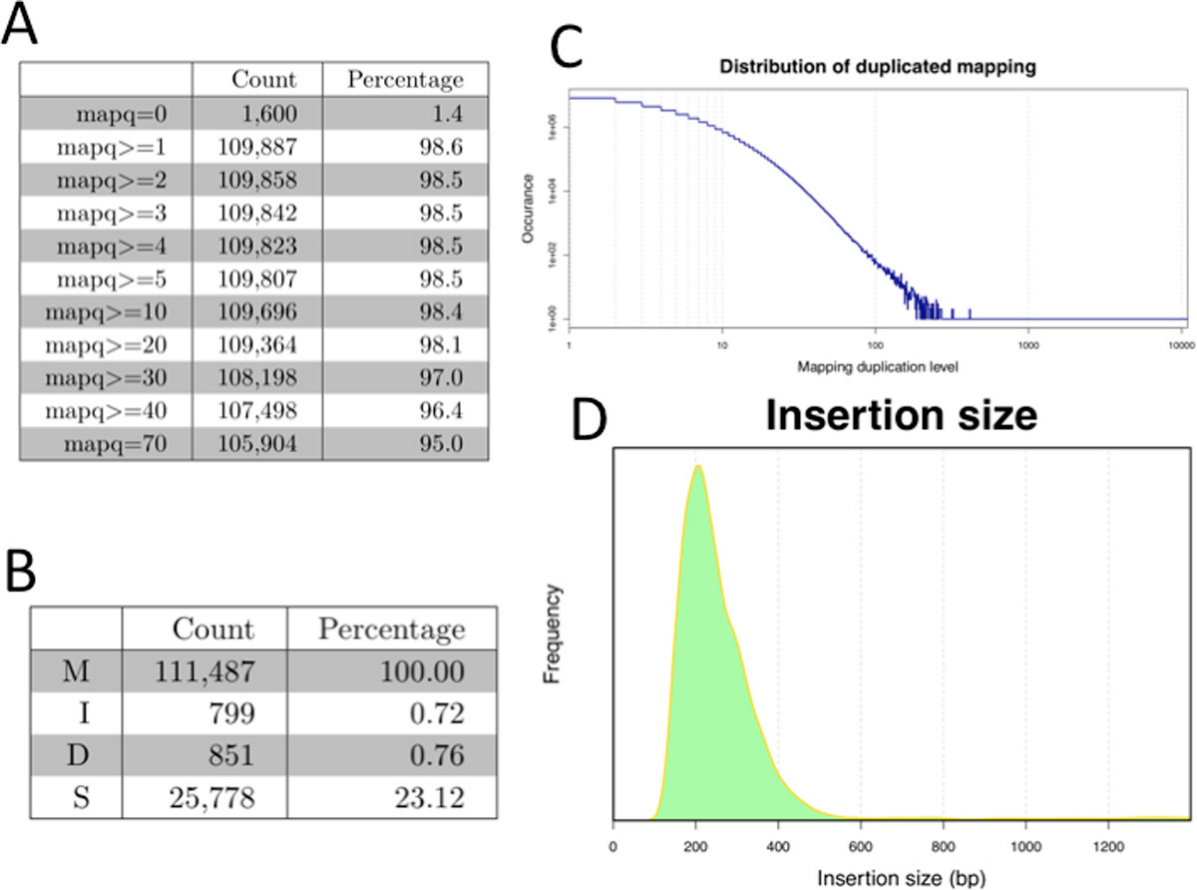
**Read mapping statistics**. (A) Map quality thresholds. The majority of reads (~95%) have the best mapping score (mapq = 70) assigned by the alignment program, suggesting high confidence of mapping results. (B) Base-level mismatch information, where M = matched bases; I = inserted bases; D = deleted bases; and S = soft clipping bases due to mismatches. (C) Duplication mapping (multiple reads mapped to the same genomic location). The x-axis represents the number of reads sharing the same mapping locations and the y-axis represents the total number of such locations. (D) Insertion size. When the BAM file includes information about paired-end reads, bamchop also summarizes the distribution of the distance between the mapped locations of the read pairs, which is known as insertion size. Insertion size equals to the size of a DNA fragment in sequencing library to be sequenced in pair.

**Figure 7:**
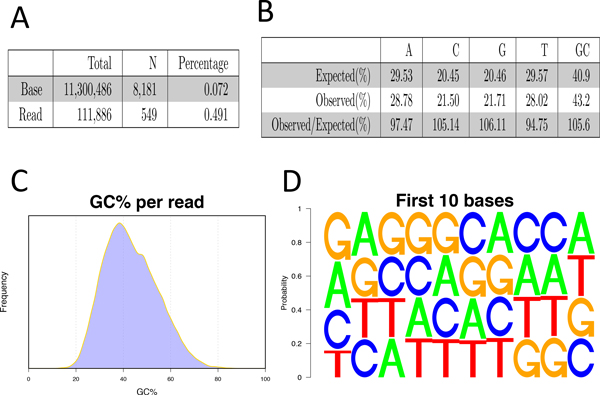
**Base frequency**. (A) Frequency of N (uncalled) bases due to low quality or ambiguity. (B) Expected versus observed base frequency. (C) Distribution of per-read GC percentages. (D) Position-specific base frequencies at the beginning of reads, which shows a bias in favor of bases G and A to start the sequencing with.

The primary output of each bamchop run is a PDF document composed of the following components:

• Overall landscape of sequencing depth. The first page of the report depicts a graphical index of sequencing depth on a chromosomal basis (Figure 3). The graphic is generated in low resolution to reduce processing time but provides a quick way to identify large genomic regions with atypical sequencing depth.

• Summary statistics. This section provides a quick review of single-value global statistics (not shown), such as the total read number of reads and the mean sequencing score.

• Read count and sequencing coverage. One of the most frequently asked questions about HTS data is whether the data provides sufficient sequencing depth to support downstream quantitative analysis. This section lists the proportions of the whole genome satisfying a number of pre-configured depth thresholds (Figure 4A), as well as the mean depth of different genomic features (Figure 4B) and chromosomes (not shown).

• Sequencing quality. Information about overall sequencing quality is essential for estimating the reliability of sequencing data. Sequencing quality usually decreases as the sequencing extends, so bases close to the end of reads are more error-prone. This section lists the proportions of bases satisfying given thresholds of quality scores (Figure 5A) as well as position-specific distributions of quality scores (Figure 5B).

• Reference genome mapping. This section summarizes the "FLAG" field of SAM format (not shown), mapping quality score (Figure 6A) assigned by the alignment program to each read, the frequency of reads with mismatches (Figure 6B), and the extent of duplicated mapping (Figure 6C). If paired-end sequencing is utilized, summary of paired mapping and distribution of insertion size is also reported (Figure 6D).

• Base frequency. Bamchop reports both frequency of Ns among all base calls and percentage of reads including any N bases (Figure 7A). Frequency of regular nucleic acid bases in sequencing reads is compared to the background frequency of bases in reference sequences (Figure 7B-C). In addition, frequency of single bases (Figure 7D), di-base combinations (not shown), and k-mers (not shown) at both ends of reads is also summarized to detect sequencing bias or primer contamination.

• Alerts. This section lists potential problems indicating low quality or suggesting adjustment of downstream data analysis. For example, an alert will be issued if the overall frequency of uncalled bases is higher than 0.5% or more than 55% of the reads are mapped to one strand.

An example of a complete bamchop report is available as Additional file 1.

### Validation

Bamchop was validated on a variety of BAM files originated from local targeted resequencing, RNA-Seq, and ChIP-Seq projects and the 1000 Genomes project. These data were generated by different sequencing machines, including Genome Analyzer IIx (Illumina Inc.), 5500 SOLiD (Life Technologies, Corp.) and 454 (Roche Diagnostics Corp.), and aligned by different programs, such as LifeScope (LifeTechnologies, Corp.), Novoalign (Novocraft Technologies), and MAQ [19].

**Figure 8:**
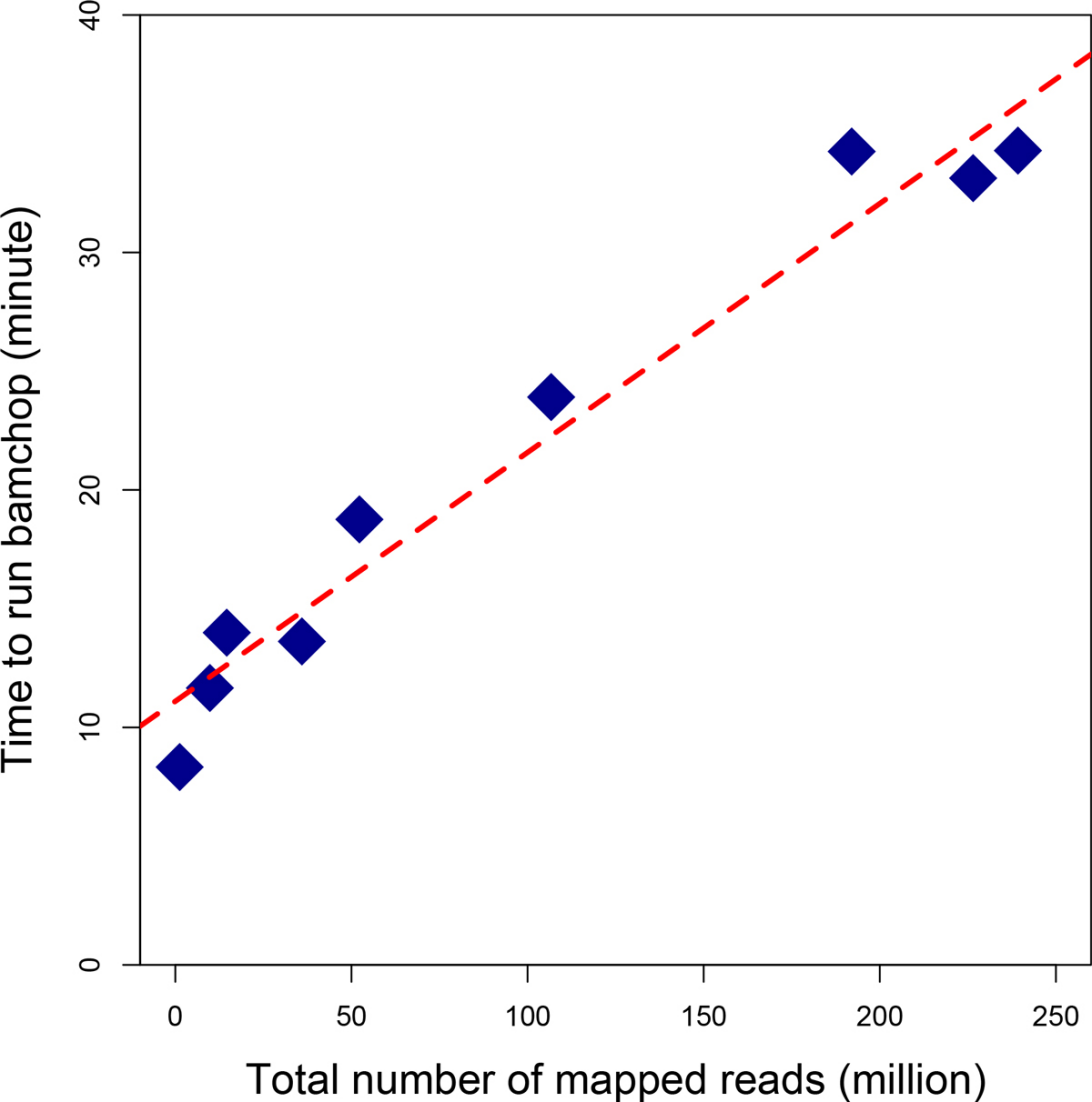
**The runtime of bamchop depends on the total number of mapped reads in each BAM file**. Diamonds represent the BAM files described in Table 1. The basic runtime of bamchop is about 11 minutes and each 100 million extra reads requires about 10.5 more minute to finish.

Part of the test runs was summarized in Table 1. Interestingly, these results showed that the runtime of bamchop had stronger correlation to the total number of mapped reads than to the size of BAM files or to the total number of mapped bases. Indeed, the total number of mapped reads and runtime significantly fit a linear regression model (p = 4X10^-6^) as shown in Figure 8. Based on this model, every 100 million extra reads in BAM files will take bamchop 10.5 more minutes to run. Therefore, bamchop is a robust and sustainable program that will be capable of handling different types and sizes of BAM files in foreseeable future.

**Table 1:** Summary of bamchop test runs

Source	Sequencer	Aligner	Paired-end	Avg. length (bp)	Total bases (million)	Total bases (billion)	BAM size (gb)	Runtime (minute)
1000 Genomes	454	MOSAIK	No	332.10	1.24	0.41	0.15	8.33
1000 Genomes	454	ssaha	No	390.00	9.81	3.83	3.31	11.66
CHOP project	SOLiD	Tophat	No	50.00	35.93	1.80	1.77	13.62
1000 Genomes	Illumina	maq	Yes	76.00	14.60	1.11	2.00	13.99
CHOP project	SOLiD	LifeScope	No	47.71	52.26	2.49	4.09	18.76
1000 Genomes	Illumina	maq	Yes	51.00	106.70	5.44	8.25	23.91
CHOP project	Illumina	Novoalign	Yes	92.92	226.50	21.05	28.89	33.13
1000 Genomes	Illumina	maq	Yes	37.00	192.02	7.10	11.10	34.25
CHOP project	Illumina	Novoalign	Yes	93.34	239.21	22.33	30.73	34.29

BAM files generated by local projects or 1000 Genome project using different sequencing instruments and alignment programs were tested with bamchop. The average mapping length, total number of aligned reads and bases, BAM file size, and time taken to finish each run were summarized in this table, which showed that the number of reads is the primary factor determining runtime.

## Conclusions

We developed a user-friendly software for biomedical researchers to rapidly and intuitively assess HTS data. The robustness of this software has been validated on BAM files of various sizes and generated by a variety of HTS experimental paradigms and sequencing workflows. Bamchop is being implemented as a core component of a workflow our group has developed for identification of sequence variations in clinical diagnostics and research samples via targeted resequencing technologies. We plan to continuously improve the functionalities of bamchop with new features and faster performance. Specifically, we plan to expand bamchop with individual modules that summarize information related to specific HTS applications, such as RNA-Seq and ChIP-Seq. Additional new functions will also include detailed information about selected genomic regions of interest and comparison of multiple BAM files.

## Availability and requirements

Contact: zhangz@email.chop.edu

Source code repository: https://github.com/CBMi-BiG/bamchop

System requirements: Unix system with at least 32 GB of RAM

Software dependency: R/Bioconductor and LaTeX documentation system

License: free for academic use

## Competing interests

The authors declare that they have no competing interests.

## Authors' contributions

ZZ designed and programmed the software, and drafted the manuscript. JL and PSW revised the manuscript. PSW oversaw the project. JL provided the original Sweave template and support to developing environment. AMY tested the program. Other co-authors participated in designing and testing the program.

## Supplementary Material

Additional file 1 **This PDF file is an example of bamchop report**. It was generated from a whole exome sequencing sample. Click here for file

## Declarations

The publication costs for this article were funded by the David Lawrence Altschuler Chair in Genomics and Computational Biology to Dr. Peter White.

This article has been published as part of BMC Bioinformatics Volume 14 Supplement 11, 2013: Selected articles from The Second Workshop on Data Mining of Next-Generation Sequencing in conjunction with the 2012 IEEE International Conference on Bioinformatics and Biomedicine. The full contents of the supplement are available online at http://www.biomedcentral.com/bmcbioinformatics/supplements/14/S11.
