# Supplementary material for: Efficient digest of high-throughput sequencing data in a reproducible report
Source: BMC Bioinformatics. 2013 Sep 13;14(Suppl 11):S3. doi: 10.1186/1471-2105-14-S11-S3 (PMC3846741; doi:10.1186/1471-2105-14-S11-S3)
Supplement: Additional file 1 — This PDF file is an example of bamchop report. It was generated from a whole exome sequencing sample. [file 1471-2105-14-S11-S3-S1.pdf]

Summarization of BAM file by bamchop: Test10

Prepared by Zhe Zhang

October 31, 2012

Contents

1 Metadata **2**

1.1 BAM file . . . 2

1.2 Summary statisitics . . . 2

2 Read count and sequencing coverage **3**

2.1 Depth categories . . . 3

2.2 By genomic feature . . . 3

2.3 By chromosome . . . 3

3 Sequencing quality **5**

3.1 Categories . . . 5

3.2 Overall distribution . . . 5

3.3 Position-specific quality score . . . 6

4 Mapping to reference genome **7**

4.1 Mapping flag . . . 7

4.1.1 Categories . . . 7

4.1.2 Flag value breakdown . . . 7

4.2 Mapping score . . . 8

4.2.1 Categories . . . 8

4.2.2 Overall distribution . . . 9

4.3 Mismatch (CIGAR) . . . 9

4.4 Duplicated mapping . . . 9

4.4.1 Categories . . . 10

4.4.2 Overall distribution . . . 11

4.5 Paired reads . . . 11

4.5.1 Total count . . . 11

4.5.2 Insertion size . . . 11

5 Base frequency **12**
5.1 N frequency 12
5.2 Expected vs. observed 12
5.3 GC content 13
5.4 Position-specific frequency 13
5.4.1 Single base 13
5.4.2 First two bases 14
5.4.3 5-mer frequency 14

6 Alerts **15**

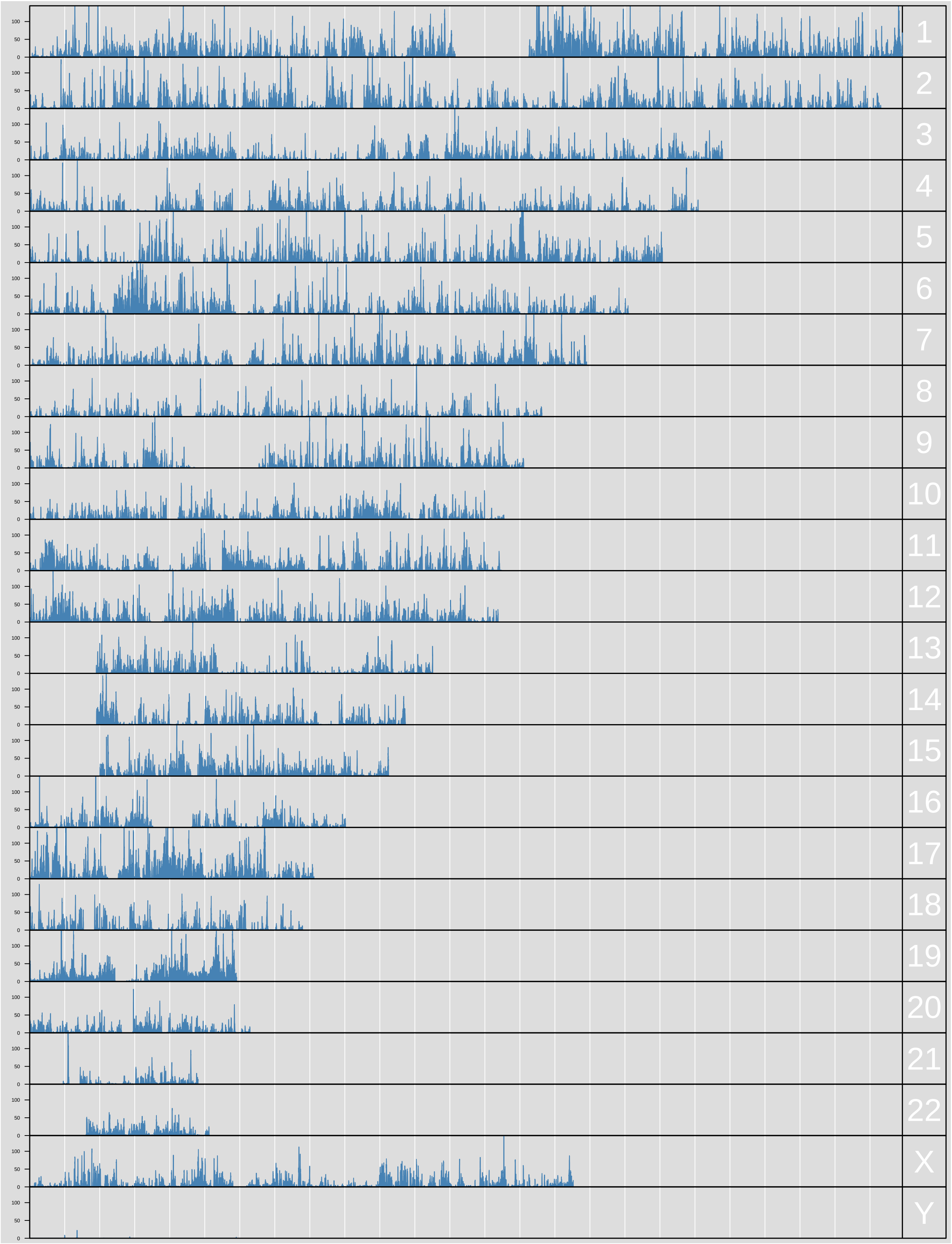
1
2
3
4
5
6
7
8
9
10
11
12
13
14
15
16
17
18
19
20
21
22
X
Y
100
50
0

1 Metadata

Sample name: Test10

Genome name: hg19

1.1 BAM file

Size: 24.31 GB

Created: 2012-01-31 15:19:17

Modified: 2012-01-26 07:31:44

Location: > nas > is1 > bamchop.1.0 > Test10.bam

1.2 Summary statisitics

Number of chromosomes	24
Total reference size (bp)	3,095,677,412
Total effective size (bp)	2,861,343,702
Total reads	354,656,714
Total mapped reads	350,525,792
Total upmapped reads	4,130,922
Forward%	50.56
N%	0.072
GC%	43.2
Duplicated%	94.7
Best sequencing quality	40
Average sequencing quality	29.69
Maximum mapped length (bp)	101
Minimum mapped length (bp)	101
Average mapped length (bp)	101
Best mapping quality	70
Average mapping quality	67.86
Highest sequencing depth	54,879
Average sequencing depth	12.37
Mapped reads per kilobase	122.5

Table 1: Summary statistics
Effective genome size excludes assembly gaps.
Sequencing quality scores are assigned by resequencer to represent base calling confidence.
Mapping quality scores are assigned by alignment program to represent mapping confidence.
Duplicates are reads mapped to the same strand and first base location as any other reads.

2 Read count and sequencing coverage

This section summarizes the coverage of reference genome by the sequencing reads. Numbers were broken down to different depth cutoffs, chromosomes, and genomic features, such as exon and intron.

2.1 Depth categories

Depth	Count	Percentage
Depth=0	1,904,563,324	66.56
Depth>=1	956,769,282	33.44
Depth>=5	314,786,636	11.00
Depth>=10	152,684,414	5.34
Depth>=20	106,411,530	3.72
Depth>=30	94,659,977	3.31
Depth>=50	81,745,011	2.86
Depth>=100	63,774,569	2.23
Depth>=1000	6,356,094	0.22
Depth>=10000	5,189	0.00

Table 2: **Depth by cutoffs.** Number and percentage of genomic locations reaching each cutoff value of sequencing depth.

2.2 By genomic feature

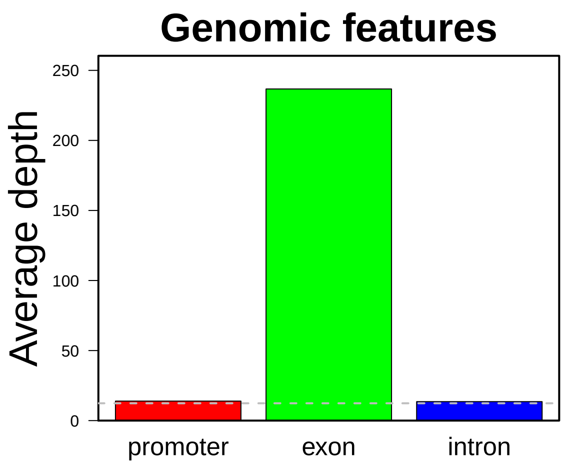

Figure 1: The average sequencing depth of different genomic features. Many applications of high-throughput sequencing technologies, such as exome sequencing and RNA-seq, expect higher depth at exons. Dahsed line indicates genomic average.

2.3 By chromosome

Table 3: Sequencing depth by chromosome

Chromosome	Chromosome_length	E↑ecitive_size	Maximum_depth	Average_depth
1	249,250,621	225,280,621	24,551	18.30
2	243,199,373	238,207,373	12,843	13.81
3	198,022,430	194,797,140	7,114	10.26
4	191,154,276	187,661,676	54,879	8.86
5	180,915,260	177,695,260	22,600	13.05

6	171,115,067	167,395,067	15,747	13.31
7	159,138,663	155,353,663	36,340	12.65
8	146,364,022	142,888,922	12,841	7.89
9	141,213,431	120,143,431	13,635	12.50
10	135,534,747	131,314,747	9,025	10.15
11	135,006,516	131,129,516	9,287	13.20
12	133,851,895	130,481,895	10,665	13.69
13	115,169,878	95,589,878	11,233	9.66
14	107,349,540	88,289,540	10,201	11.47
15	102,531,392	81,694,769	13,853	13.86
16	90,354,753	78,884,753	7,449	11.21
17	81,195,210	77,795,210	10,513	22.87
18	78,077,248	74,657,248	8,070	10.34
19	59,128,983	55,808,983	9,983	22.64
20	63,025,520	59,505,520	17,934	9.51
21	48,129,895	35,108,702	8,045	7.03
22	51,304,566	34,894,566	12,420	10.39
X	155,270,560	151,100,560	10,073	9.23
Y	59,373,566	25,653,566	3,839	0.17

3 Sequencing quality

This section summarizes the quality score assigned by the sequencing machine to each base of each read.

Quality score summary:

```
Min. 1st Qu.  Median    Mean 3rd Qu.    Max.
1.00   30.00   31.00   29.69   31.00   40.00
```

3.1 Categories

Score	Count	Percentage
Score=0	0	0.00
Score>=5	11,071,484	97.97
Score>=10	11,029,282	97.60
Score>=13	10,991,451	97.27
Score>=20	10,914,943	96.59
Score>=30	9,496,997	84.04
Score>=40	1	0.00

Table 4: This table lists the number and frequency of sequencing quality scores above given cutoff values.(TODO: add a column for score p values.)

3.2 Overall distribution

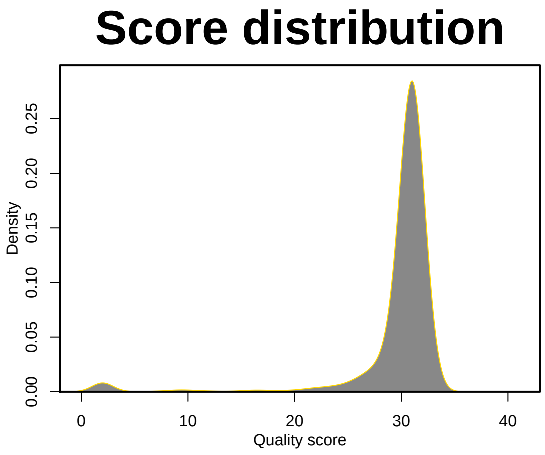

Figure 2: This distribution is based on all bases of randomly selected sequencing reads, so position-specific sequencing quality is not considered (see next section). The quality scores are calculated by subtracting 33 after converting ASCII characters in BAM file to integers. If the convention of Sanger sequencing was followed, they are equal to Phred scores.

3.3 Position-specific quality score

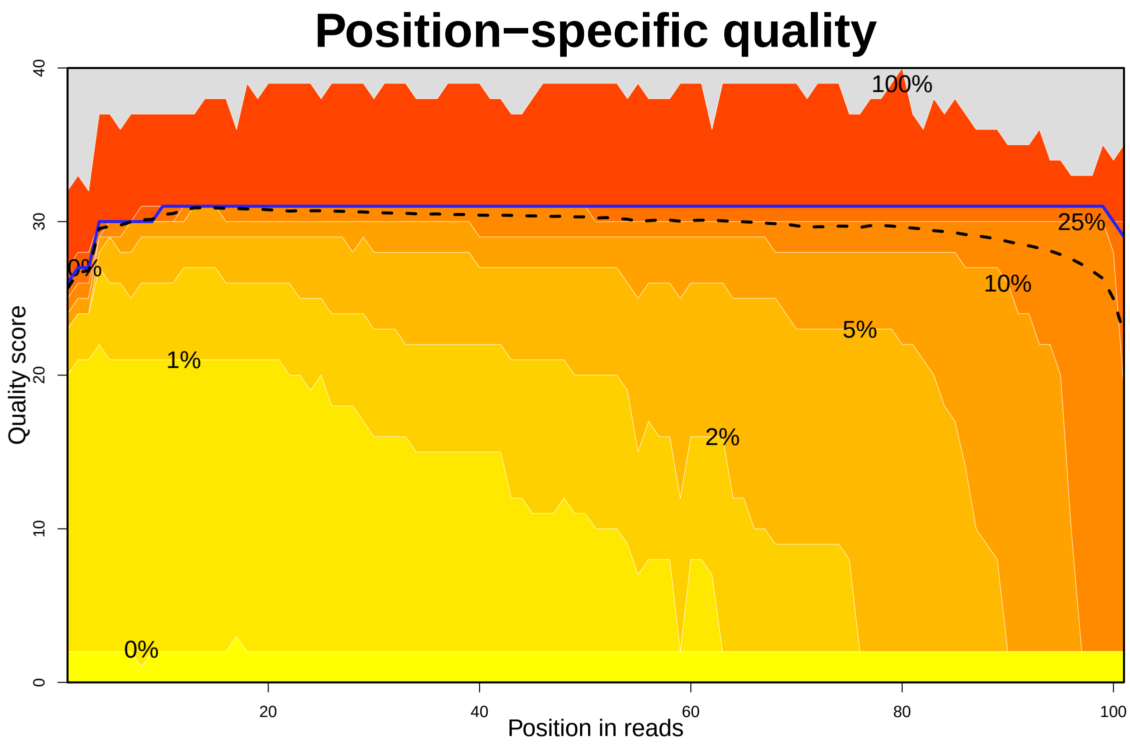

Figure 3: This plot shows quality scores at different positions within reads. The blue and dashed lines represent the median and mean basecall qualities, respectively. The heat gradient corresponds to percentiles.

4 Mapping to reference genome

This section summarizes the information related to the mapping of reads to reference genome.

Mapping length:

```
   Min. 1st Qu.  Median    Mean 3rd Qu.    Max.
    101     101     101     101     101     101
```

4.1 Mapping flag

Mapping flag is a field of SAM format using a series of bitwise codes to represent different combinations of mapping results:

Bitwise	Description
0X1	template having multiple segments in sequencing
0X2	each segment properly aligned according to the aligner
0X4	segment unmapped
0X8	next segment in the template unmapped
0X10	SEQ being reverse complemented
0X20	SEQ of the next segment in the template being reversed
0X40	the first segment in the template
0X80	the last segment in the template
0X100	secondary alignment
0X200	not passing quality controls
0X400	PCR or optical duplicate

4.1.1 Categories

Code	Count	Percentage
0X1	354,656,714	100.00
0X2	330,661,584	93.23
0X4	4,130,922	1.16
0X8	4,130,922	1.16
0X10	175,338,322	49.44
0X20	175,338,982	49.44
0X40	177,328,483	50.00
0X80	177,328,231	50.00
0X100	0	0.00
0X200	0	0.00
0X400	0	0.00

Table 5: **Flag categories.** The total number and percentage of flagged reads in each category.

4.1.2 Flag value breakdown

Table 6: The breakdown of values into categories. All mapped and unmapped reads were counted.

Value	Count	Percentage	0X1	0X2	0X4	0X8	0X10	0X20	0X40	0X80	0X100	0X200	0X400
65	2,018,299	0.57	X	-	-	-	-	-	X	-	-	-	-
69	811,949	0.23	X	-	X	-	-	-	X	-	-	-	-
73	1,242,724	0.35	X	-	-	X	-	-	X	-	-	-	-
81	2,016,394	0.57	X	-	-	-	X	-	X	-	-	-	-
83	84,020,549	23.69	X	X	-	-	X	-	X	-	-	-	-
89	1,306,040	0.37	X	-	-	X	X	-	X	-	-	-	-
97	1,748,206	0.49	X	-	-	-	-	X	X	-	-	-	-
99	81,310,243	22.93	X	X	-	-	-	X	X	-	-	-	-
101	770,209	0.22	X	-	X	-	-	X	X	-	-	-	-
113	2,083,870	0.59	X	-	-	-	X	X	X	-	-	-	-
129	2,018,926	0.57	X	-	-	-	-	-	-	X	-	-	-
133	1,242,724	0.35	X	-	X	-	-	-	-	X	-	-	-
137	811,949	0.23	X	-	-	X	-	-	-	X	-	-	-
145	1,747,726	0.49	X	-	-	-	X	-	-	X	-	-	-
147	81,310,243	22.93	X	X	-	-	X	-	-	X	-	-	-
153	770,209	0.22	X	-	-	X	X	-	-	X	-	-	-
161	2,016,574	0.57	X	-	-	-	-	X	-	X	-	-	-
163	84,020,549	23.69	X	X	-	-	-	X	-	X	-	-	-
165	1,306,040	0.37	X	-	X	-	-	X	-	X	-	-	-
177	2,083,291	0.59	X	-	-	-	X	X	-	X	-	-	-

4.2 Mapping score

Mapping scores are assigned by the alignment program to indicate the likelihood of false alignment and stored in the ”mapq” field of BAM files. Higher score usually means high alignment confidence.

Mapq summary:

```
Min. 1st Qu.  Median    Mean 3rd Qu.    Max.
0.00   70.00   70.00   67.86   70.00   70.00
```

4.2.1 Categories

Score	Count	Percentage
mapq=0	1,543	1.38
mapq>=1	110,343	98.62
mapq>=2	110,319	98.60
mapq>=3	110,295	98.58
mapq>=4	110,277	98.56
mapq>=5	110,257	98.54
mapq>=10	110,163	98.46
mapq>=20	109,834	98.17
mapq>=30	108,740	97.19
mapq>=40	107,980	96.51
mapq=70	106,441	95.13

Table 7: **Mapq categories.** The total number and percentage of reads having higher mapping score than given values.

4.2.2 Overall distribution

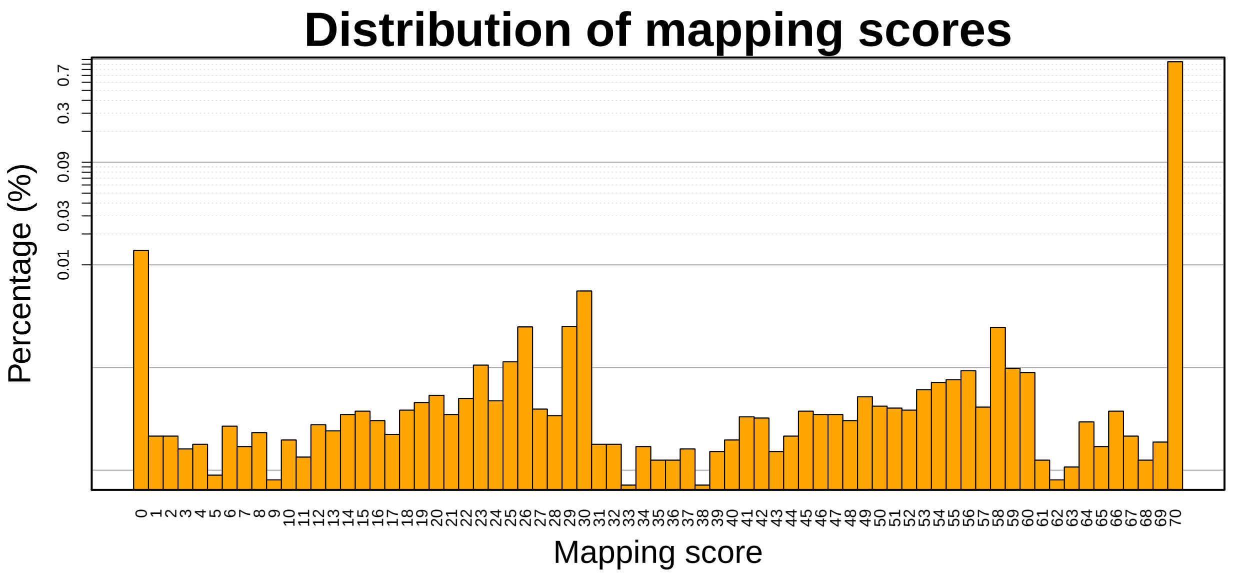

Figure 4: Mapping quality is assigned to each read by alignment program to indicate the uniqueness and fitness of the alignment. By definition, mapping quality equals to -10Log10(Prob. of incorrect mapping); however, its calculation depends on individual programs. Lower scores usually suggest more mismatches AND/OR more genomic locations to be mapped to.

4.3 Mismatch (CIGAR)

SAM uses CIGAR strings to compactly represent alignments. CIGAR characters are used in concert with lengths to describe various types of matching, mismatching, clipping, padding and splicing events within an alignment.

Bitwise	Description
M	alignment match (can be a sequence match or mismatch)
I	insertion to the reference
D	deletion from the reference
N	skipped region from the reference
S	soft clipping (clipped sequences present in SEQ)
H	hard clipping (clipped sequences NOT present in SEQ)
P	padding (silent deletion from padded reference)
=	sequence match
X	sequence mismatch

4.4 Duplicated mapping

Duplicated mapping refers to reads whose 5'-end were mapped to the same genomic location. The level of duplication is an indicator of the effect of PCR artifact, but also depends on local and overall sequencing depth.

Category	Count	Percentage
M	111,886	100.00
I	821	0.73
D	1,024	0.92
S	25,923	23.17

Table 8: **Mismatch frequency** The total number and percentage of reads with specific type of mismatches.

The average number of duplicated reads at each mapping location is 4.617.

4.4.1 Categories

Duplication_level	Occurance	Read_count	Percentage
1	8,266,440	8,266,440	5.30
2	6,056,965	12,113,930	7.76
3	4,486,411	13,459,233	8.63
4	3,351,383	13,405,532	8.59
5	2,526,440	12,632,200	8.10
6	1,917,507	11,505,042	7.37
7	1,460,183	10,221,281	6.55
8	1,121,508	8,972,064	5.75
9	865,931	7,793,379	5.00
10	679,791	6,797,910	4.36
>10	3,055,851	50,845,574	32.59

Table 9: **Categories of duplication levels.** The results in this table are based on the first 10 million reads of each chromosome mapped to the forward strand. Read count and percentage are the total number and percentage of reads falling into each category.

4.4.2 Overall distribution

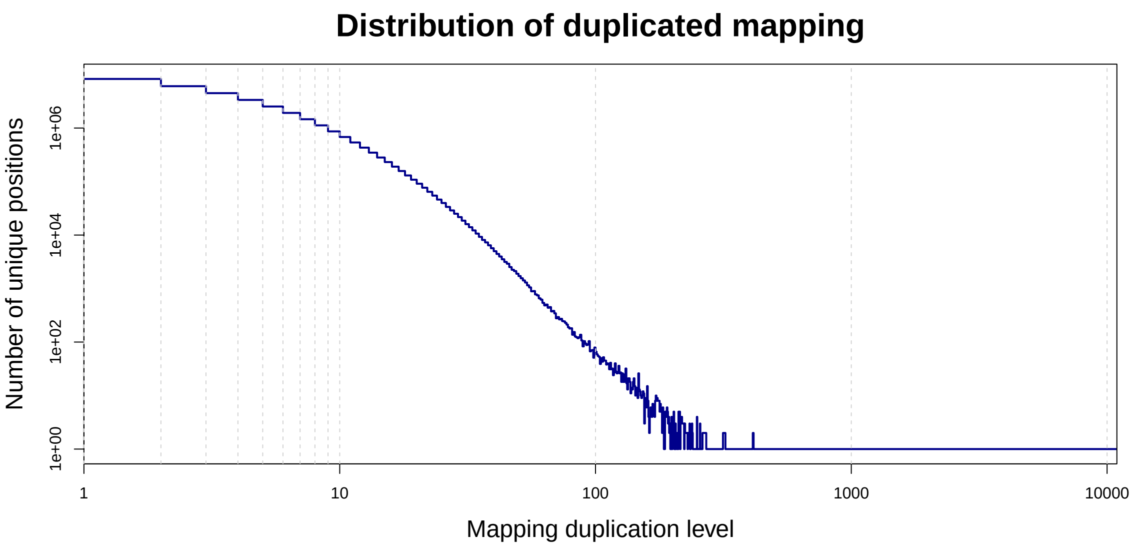

Figure 5: Duplication level of read mapping. The x-axis indicates the number of reads sharing the same mapping location of their 5'-end and the y-axis is the total occurance of each level. Only reads mapped to the forward strand and the first 10 million reads of each chromosome was used to reduce computation.

4.5 Paired reads

Information about paired-end reads is available in this BAM file.

4.5.1 Total count

Category	Count	Percentage
Total paired-end reads	354,656,714	100.00
Ends properly mapped	330,661,584	93.23
One end unmapped	4,130,922	1.16

Table 10: **Paired-end reads.** Read counts in this table are based on the "flag" field in BAM file. Properly mapping paired-end reads are reads mapped to the opposite strand of the same chromosome.

4.5.2 Insertion size

Size summary:

```
   Min.   1st Qu.    Median      Mean   3rd Qu.      Max.
     11       177       215   1320000       272 228400000
```

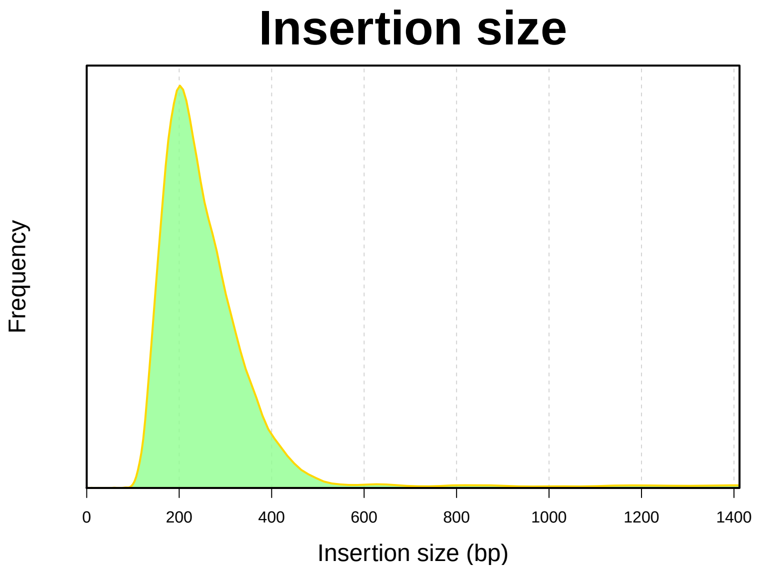

Figure 6: **Distribution of insertion size.** Insertion size is the distance between the mapping locations of the 5'-end of paired reads. It represents the size of DNA fragment to be sequenced.

5 Base frequency

This section summarizes the frequency of nucleic acid bases within sequencing reads in order to identify sequencing bias.

5.1 N frequency

	Total	N	Percentage
Base	11,300,486	8,181	0.072
Read	111,886	549	0.491

Table 11: **N base frequency.** The Ns in the reads are assigned by the sequencing machine to suggest that the base cannot be determined due to low quality or other reasons. This table shows the number and percentage of Ns and reads including any Ns. Ns are then excluded from the following analyses of base frequency.

5.2 Expected vs. observed

	A	C	G	T	GC
Expected(%)	29.53	20.45	20.46	29.57	40.9
Observed(%)	28.78	21.50	21.71	28.02	43.2
Observed/Expected(%)	97.47	105.14	106.11	94.75	105.6

Table 12: **Expected vs. observed.** The expected base frequency is based on the whole reference genome and the observed frequency is the base frequency in sequencing reads. Their ratio reflects the sequencing bias of nucleic acid bases.

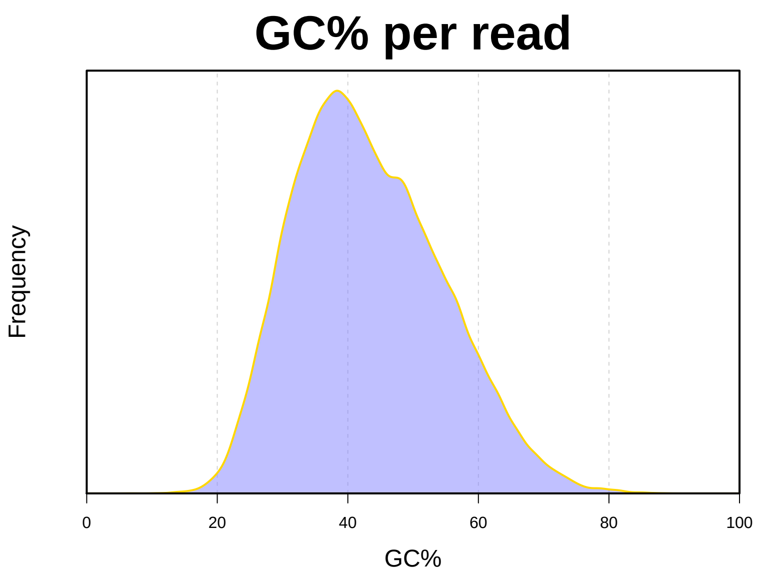

Figure 7: **GC content.** Percentage of C/G bases within each read.

5.3 GC content

5.4 Position-specific frequency

Position-specific frequency of bases indicates whether there is a sequencing bias at both ends of the reads. The bias can be introduced via a variety of sources, such as DNA fragmentation and primer contamination.

5.4.1 Single base

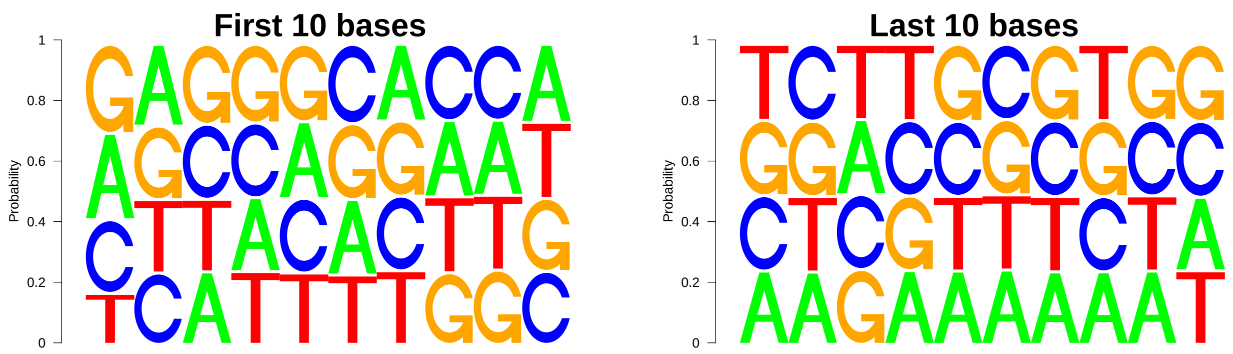

Figure 8: **Base frequency at both ends.**The base frequency of the first and last 10 bases (the rightmost is the last base) of reads. The frequency was normalized by the overall base frequency with sequencing reads, so this summary indicates the preference of sequencing to start with a given nucleic acid base.

5.4.2 First two bases

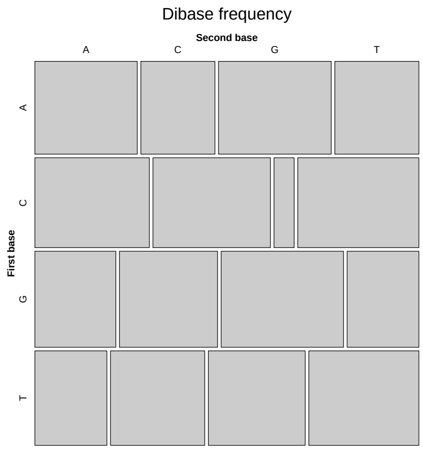

Figure 9: **First two base combination.** This plot summarizes the frequency of the two-base combinations at the 5'-end of reads. The size of the blocks represent their relative frequency after adjusted by their expected frequency based on the position-specific frequency of the first two bases.

5.4.3 5-mer frequency

Table 13: 5-mers over-represented at either end of reads.

5-mer	Expected_count	Observed_count	Observed/Expected
-TCCCA	58.51	182	3.11
-CCTCC	60.76	191	3.14
-TGGGA	69.96	220	3.14
GGGAG-	75.22	237	3.15
-CTGGC	73.99	235	3.18
-GCCTC	74.91	241	3.22
TGGGG-	76.11	245	3.22
-TCCCT	52.93	172	3.25
-CCCTG	63.90	209	3.27
-CTCTG	85.77	285	3.32
TTTTT-	196.17	658	3.35
-CTCCC	64.61	220	3.41
-CTGGG	77.58	267	3.44
AAAAA-	196.59	683	3.47
-TTTTT	113.58	395	3.48
CCTCC-	70.60	246	3.48
-GGCTG	82.01	286	3.49
-GCCTG	78.55	278	3.54
CTCCC-	69.62	250	3.59
CTGGG-	74.45	273	3.67
-GCTGG	83.19	307	3.69
-GGAGG	88.83	330	3.72
-CCCAC	63.45	236	3.72
GGAGG-	74.61	292	3.91
-TGGGG	53.39	215	4.03
-CCCAG	66.53	275	4.13
GGGGG-	59.06	259	4.39

6 Alerts

— Less than 1% (0.032%) of the total reads were randomly selected to summarize sequencing quality, mapping quality, duplicated mapping, mismatch frequency and base frequency.
— The average sequencing quality is less than 30 (29.69).
— More than 80% (95%) of the mapped reads share the same mapping locations with other reads (duplication).
— There are 27 5-mers overrepresented at either end of reads.
